# Adipose Tissue-Derived Stem Cells Reduce Acute and Chronic Kidney Damage in Mice

**DOI:** 10.1371/journal.pone.0142183

**Published:** 2015-11-13

**Authors:** Marina Burgos-Silva, Patricia Semedo-Kuriki, Cassiano Donizetti-Oliveira, Priscilla Barbosa Costa, Marco Antonio Cenedeze, Meire Ioshie Hiyane, Alvaro Pacheco-Silva, Niels Olsen Saraiva Câmara

**Affiliations:** 1 Nephrology Division, Federal University of São Paulo, São Paulo, São Paulo, Brazil; 2 Department of Immunology-Institute of Biomedical Sciences IV, University of São Paulo, São Paulo, São Paulo, Brazil; University of Torino, ITALY

## Abstract

Acute and chronic kidney injuries (AKI and CKI) constitute syndromes responsible for a large part of renal failures, and are today still associated with high mortality rates. Given the lack of more effective therapies, there has been intense focus on the use stem cells for organ protective and regenerative effects. Mesenchymal stem cells (MSCs) have shown great potential in the treatment of various diseases of immune character, although there is still debate on its mechanism of action. Thus, for a greater understanding of the role of MSCs, we evaluated the effect of adipose tissue-derived stem cells (AdSCs) in an experimental model of nephrotoxicity induced by folic acid (FA) in FVB mice. AdSC-treated animals displayed kidney functional improvement 24h after therapy, represented by reduced serum urea after FA. These data correlated with cell cycle regulation and immune response modulation via reduced chemokine expression and reduced neutrophil infiltrate. Long-term analyses, 4 weeks after FA, indicated that AdSC treatment reduced kidney fibrosis and chronic inflammation. These were demonstrated by reduced interstitial collagen deposition and tissue chemokine and cytokine expression. Thus, we concluded that AdSC treatment played a protective role in the framework of nephrotoxic injury via modulation of inflammation and cell cycle regulation, resulting in reduced kidney damage and functional improvement, inhibiting organ fibrosis and providing long-term immune regulation.

## Introduction

Acute kidney injury (AKI) is a major health concern with a period prevalence varying between 1.4% to 25.9%, committing approximately 5,7% of intensive care patients, and contributing with an overall hospital mortality rate of an estimated 60.3% of AKI patients [1]. Amongst the different types of acute kidney injury (AKI), nephrotoxic damage promoted by radiocontrast media, antibiotics, nonsteroidal anti-inflammatory drugs (NSAIDs) and immunosuppressants such as cyclosporine are major contributors of kidney function impairment. In addition, statistics indicate that 19% of all renal injuries are related to nephrotoxic events [1]. Physiologically, AKI manifests as a sudden impairment of kidney function. At a cellular level, kidney tissue is taken by an inflammatory process and inflammatory immune cell infiltration. This environment amplifies kidney damage and worsens kidney function. This inflammatory phase eventually resolves, leaving the organ with near optimal function. More recent studies indicate that after AKI, kidneys enter a steady and often subclinical progression towards chronic kidney injury (CKI), with fibrogenesis and eventual organ failure [2,3]. A well-characterized murine model of AKI has been developed through the administration of high doses of folic acid (FA), with prominent tissue inflammation and cell cycle deregulation [4–6]. In addition to its acute renal damage, studies show that this injury also progresses to CKI, making it a good model towards the understanding of progression to chronic fibrosis [7,8].

Stem cells have been designated as a potential therapeutic tool in the treatment of diverse inflammation-based pathologies, including AKI and CKI. Indeed, adipose tissue-derived stem cell (AdSC) therapy has also shown to reduce tissue inflammation and kidney injury in rodent models of AKI [9,10]. However, the long-term consequences to stem cell treatment are little known. Previous data published in our laboratory suggest that this treatment may reduce the progression to CKI in ischemia/reperfusion injury [11,12], though, to date, few studies have evaluated this effect in drug-induced kidney injury [13,14]. This study aimed to elucidate the potential role of AdSC in the treatment of folic acid-induced AKI and its long-term repercussions in disease progression. Results showed stem cell treatment reduced acute kidney damage with ameliorated kidney function and reduced inflammation. Moreover, this intervention also led to reduced fibrosis and long-term immunomodulatory effects in kidney tissue, demonstrating this cells potential to promote both short and long-term beneficial effects after kidney damage.

## Methods

### Animal model and sample preparation

Male FVB mice (8 weeks aged) were obtained from CEDEME (Federal University of São Paulo, São Paulo, Brazil). Animal procedures followed the UNIFESP Research Ethics Committee Guidelines and the study was approved by the Brazilian institutional animal care and use committee (CEUA) (CEP process no. 602/2010). Mice were kept under a 12 hour light-dark cycle at a temperature of 23–27°C with water and chow *ad libitum*. In order to induce kidney injury, FVB/NJ mice received folic acid (200 mg/kg) in NaHCO_3_ (0.15 M) vehicle i.p. according to the protocol established by Klingler *et al*. adapted by our laboratory. At the determined time, animals were killed by anesthetic overdose of xilazine /ketamine (Agribands, Brazil) to obtain kidney and blood samples. Blood samples were used for kidney functional analysis. Kidneys fragments were snapped frozen in liquid nitrogen for gene expression assays or fixed in buffered formalin and embedded in paraffin for immunohistological assays.

### Kidney function

Serum urea levels were evaluated through colorimetric assays based on the urease method using deproteinized serum samples (Labtest, Brazil). Values were determined using a Synergy Mx spectrophotometer (Biotek, Winooski, Vermont, USA) and analyzed accordingly.

### Stem Cells

Culture: AdSC were obtained through Collagenase IA digestion of inguinal adipose tissue from FVB male mice and posterior culture in Dulbecco´s Eagle medium (DMEM) low glucose (Cultilab, Brasil) supplemented with 10% fetal bovine serum (FBS) (Gibco,USA) and maintained for a maximum of 5 passages at 5% CO_2_, 37°C. Stem cell profile was evalued through membrane receptor phenotyping and differentiation assays. Stem cell markers were determined through cytometric analysis for CD34, CD45, CD73, CD90 and CD105 (BD Biosciences, USA), FACSCalibur flow cytometer (BD Biosciences, San Jose, California, USA). Stem cell pluripotency was evaluated by culture osteogenesis and adipogenesis under specific differentiation medium stimuli. Osteogenesis was induced with low glucose DMEM supplemented with 10% FBS, dexamethasone (0.1 μM), ascorbic acid (0.2 mM), and beta glycerol phosphate (10 mM) (Sigma, USA) for 24 days. Adipogenesis was induced with high glucose DMEM medium supplemented with 10% FBS, dexamethasone (1 μM), isobutylmethylxanthine (0.5 mM), insulin (10 μg/ml), and indomethacin (100 μM) (Sigma) for 18 days. Adipocyte lipid and osteocyte calcium deposits were stained with Oil Red and Von Kossa or Alizarin Red (Chemicon, USA), respectively. Stem cell treatment: Cultured AdSCs expanded through a maximum of 5 passages were suspended in sterile phosphate buffered saline (PBS) and administered intraperitoneally into FVB mice (1x10^6^ cells per animal) 24h after folic acid.

### Histology and Immunohistochemistry

In order assess kidney regeneration, paraffin-embedded sections were submitted to immunohistochemistry after heat mediated antigen retrieval and stained for Proliferating cell nuclear antigen (PCNA, clone PC10) (DAKO, USA) or Myeloperoxidase (MPO) (DAKO, USA), using a Horseradish Peroxidase (HRP)-conjugated secondary antibody EnVision^™^+ Dual Link System-HRP (Dako, EUA) revealed with 3,3’-diaminobenzidine (DAB)+ substrate-chromogen (Dako, EUA). In order to evaluate tissue fibrogenesis: Paraffin-embedded sections were submitted to picrosirius staining and subsequent polarization microscopy in order to distinguish collagen fibers. Kidney section images were captured at room temperature (21–25°C) at approximately 10 fields per kidney using an Olympus BX60 microscope and NIS-Elements F capture system (Nikon, Center Valley, PA, USA) or Leica DM 1000 microscope and Leica DFC310 FX (Leica, Wetzlar, Germany) capture system. Positive tissue staining was quantified through the use of Leica Application Suite (Leica, Wetzlar, Germany) or NIS-Elements AR (Nikon, Center Valley, PA, USA) software.

### Bio-plex

A Bio-Plex mouse cytokine assay kit (Bio-Rad, USA) was used to test samples for the presence cytokines in kidney tissue. The assay was read on the Bio-Plex suspension array system (Bio-Rad Laboratories, Inc., Hercules, CA, USA), and data was analyzed using Bio-Plex Manager software version 4.0. Standard curves ranged from 32,000 to 1.95 pg/ml.

### Statistic analysis

Data presented are expressed as mean ± Standard Deviation (s.d.). Differences between experimental groups were evaluated for statistical significance using ANOVA followed by the Bonferroni test and Student’s t-test. Differences were considered significant at *P*< 0.05.

## Results

### AdSCs characterization

**Fig 1:**
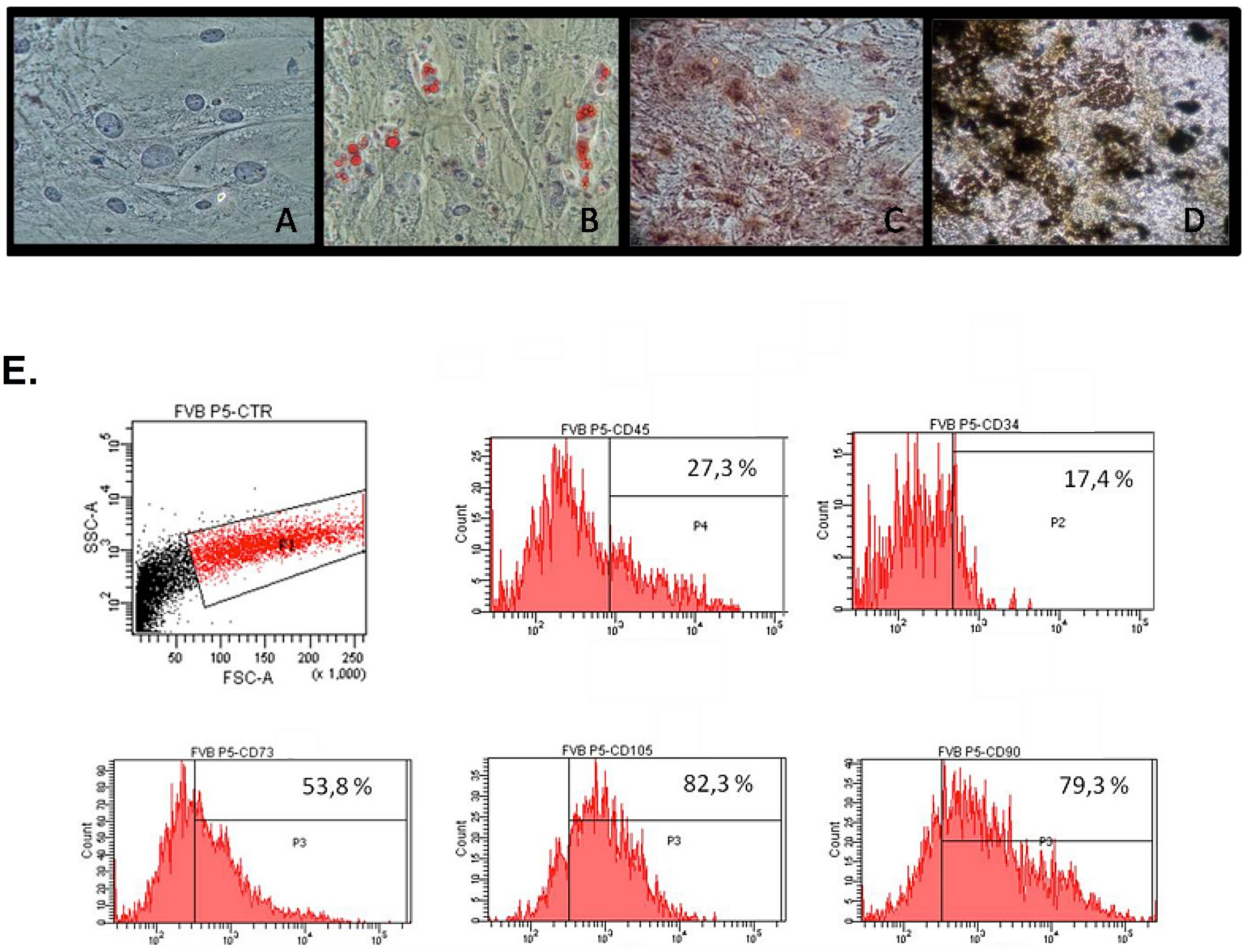
Stem cell characterization. AdSC from FVB mice were maintained for a maximum of 5 passages and stem cell profile was evaluated through membrane receptor phenotyping and differentiation assays. **(A)** Representative image of AdSC displaying cell phenotype under bright-field microscopy (original magnification: 20x). **(B-D)** Stem cell pluripotency was evaluated by culture adipogenesis and osteogenesis under differentiation stimuli and posterior staining with (**B)** Oil Red and (**C)** Alizarin Red or (**D)** Von Kossa, respectively (original magnification: 20x). **(E)** Representative histograms for stem cell marker expression (CD34, CD45, CD73, CD90 and CD105) obtained by cytometric analysis.

FVB AdSCs were initially expanded for 5 passages and later characterized by cell differentiation and immunophenotyping assays. Cultured cells displayed typical fibroblastoid morphology (Fig 1A) and under appropriate stimuli, exhibited potential for adipocyte and osteocyte differentiation demonstrated through Oil Red, Alizarin and Von Kossa staining (Fig 1B–1D). Cells also expressed characteristic stem cells markers CD73, CD105 and CD90, being negative for CD45 and CD34 (Fig 1E).

### AdSCs ameliorate folic acid-induced AKI

**Fig 2:**
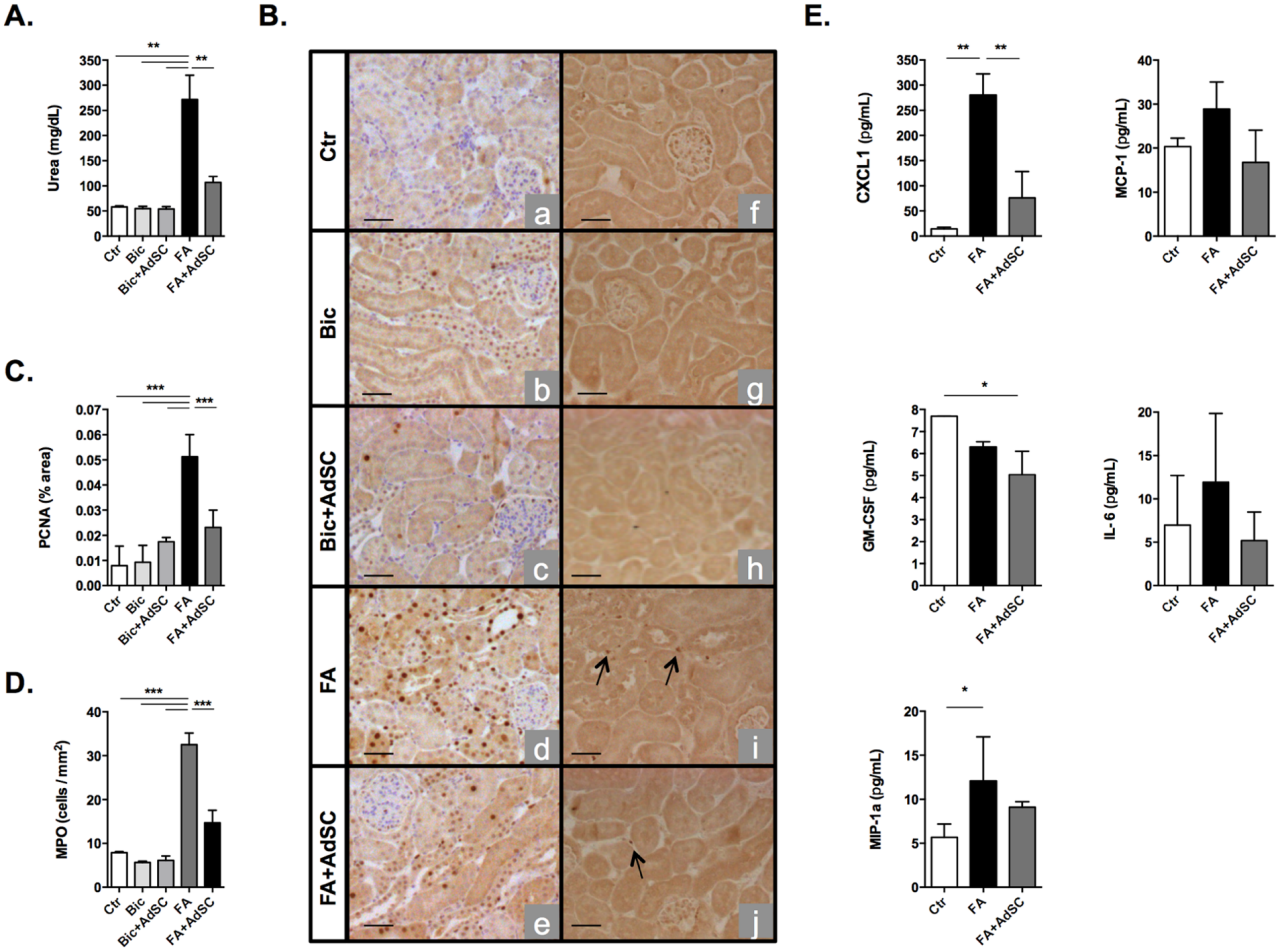
Folic Acid-induced acute kidney injury. Mice were submitted to folic acid-induced kidney injury (FA) or sodium bicarbonate (Bic) vehicle treatment and after 24 h, received adipose tissue-derived stem cells (AdSC). Kidney tissue and serum samples were extracted 24 h after AdSC therapy for kidney function and protein expression analyses; control (ctr) mice received no treatment, (Bic) mice received vehicle only (sodium bicarbonate, 0.15 M). **(A)** Urea biochemistry essays revealed that AdSC treatment conferred protection against acute kidney dysfunction as seen through lower serum urea accumulation (n = 2–6 for each group); **(B-D)** Immunohistochemistry of kidney tissue. Semi-quantification of proliferating cell nuclear antigen (PCNA) staining demonstrates normalized cell proliferation rates after AdSC treatment vs. FA mice (B,C) (n = 2–5 for each group). Immunohistochemistry also showed reduced neutrophil infiltrate seen through myeloperoxidase (MPO) staining after AdSC treatment vs. FA mice (B,D) (n = 2–3 for each group). Arrows indicate positive MPO staining (original magnification: 20x). **(E)** Kidney bioplex essays showed reduced inflammatory profiles in stem cell treated mice, (n = 3 for each group). Neutrophil chemoattractant protein chemokine (C-X-C motif) ligand 1 (CXCL1), and macrophage inflammatory protein 1 alpha (MIP-1**α**) levels n were increased in AdSC-treated mice. In parallel, granulocyte macrophage colony stimulating factor (GM-CSF) and CXCL-1 expression was reduced in the AdSC-treated group. A tendency towards lower monocyte chemoattractive protein-1 (MCP-1) and interleukine 6 (IL-6) expression was also observed for these animals (n = 2–3 for each group). Serum urea levels are expressed as (mg/dL ± s.d.). Immunohistochemistry values are expressed as the percentage of area positive for PCNA ± s.d. or the number of cells positively stained for MPO per mm^2^ ± s.d.). Scale bar, 25 **μ**m. Bioplex values are expressed as (pg/mL) ± s.d. *P<0.05, **P<0.01 ***P<0.001

In order to evaluate the effect of AdSC treatment on a mouse model of nephrotoxic kidney injury, our studied initially focused on the acute effects of this therapy on FA-induced AKI. For so, FVB mice received 200 mg/kg FA (i.p.) and after 24 h were submitted to singenic AdSC therapy. Initial essays indicated that FA significantly impaired kidney function, inducing the accumulation of serum urea. In contrast, stem cells protected kidneys against AKI, as seen through a reduction in serum urea levels 48 h after FA (Fig 2A).

Kidney tubule cells are the principal cell targets of FA toxicity [7,15,16]. Many studies involving experimental kidney models describe FA as being a potent mitogenic factor, inducing renal tubule cell irregular proliferation [17]. We observed abnormal tubule cell expression of proliferating cell nuclear antigen (PCNA) through immunohistochemistry, indicating a dysregulated cell cycle of these cells. In addition, AdSC treatment reduced this profile to basal levels, maintaining these cells in their original state (Fig 2B and 2C).

A major focus in AKI is the inflammation and immune cell infiltration that takes effect due to tissue injury. Since neutrophil expression in kidney tissues MPO expression in the AdSC-treated group was significantly reduced in kidneys after FA-induced injury in comparison to non-treated animals (Fig 2B and 2D). In order to verify the inflammatory milieu developed in kidneys recruitment into damaged tissue is a crucial step in tissue injury induced by FA, we investigated myeloperoxidase (MPO), we performed bioplex proteomic studies for chemokines and inflammatory cytokines. There was a significant rise in the expression of Chemokine (C-X-C motif) ligand 1 (CXCL-1) and macrophage inflammatory protein 1alpha (MIP-1**α**) due to FA injury with a reduction of CXCL-1 levels in AdSC-treated animals in comparison to the latter and a reduction of Granulocyte-macrophage colony-stimulating factor (GM-CSF) levels in comparison with control (Fig 2E).

### AdSC-treatment prevents folic acid-induced CKI

**Fig 3:**
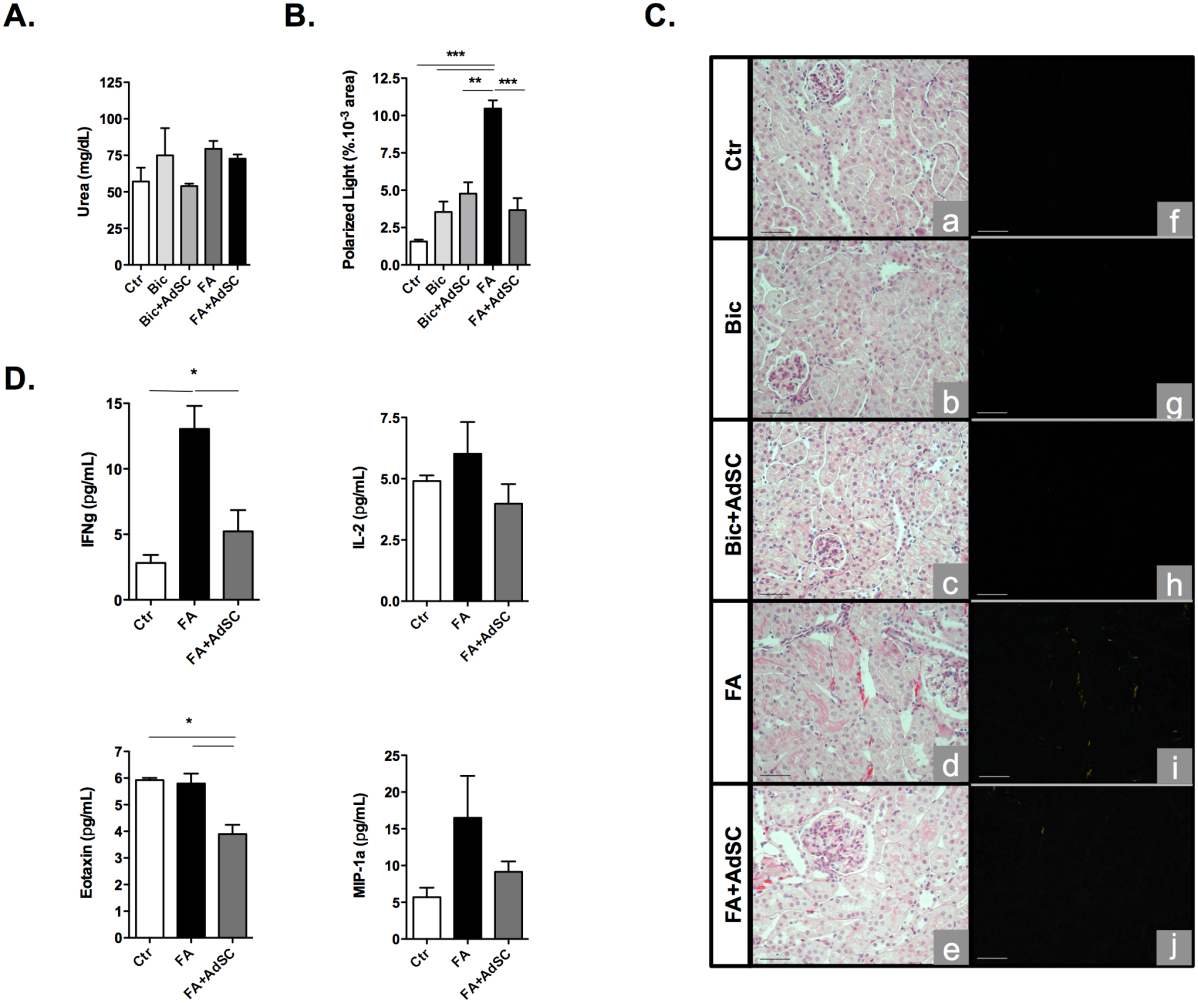
Folic Acid-induced chronic kidney injury. Mice were submitted to folic acid-induced kidney injury (FA) or sodium bicarbonate (Bic) vehicle treatment and after 24 h, received adipose tissue-derived stem cells (AdSC). Kidney tissue and serum samples were extracted 4 weeks after FA for kidney function and protein expression analyses; control (Ctr) mice received no treatment, (Bic) mice received vehicle only (sodium bicarbonate, 0.15 M). **(A)** Urea biochemistry essays showed no significant difference of long term renal functional between groups (n = 2–7 for each group) **(B,C)** Kidney sections were viewed under both bright-field (a-e) and polarized light (f-j) microscopy. AdSC-treated mice kidneys displayed reduced deposit of tissue collagen in comparison to FA mice (n = 3–5 for each group) (original magnification: 20x). **(D)** Bioplex essays show stem cell-treated mice develop ameliorated chronic kidney inflammation (n = 3 for each group). FA-treated mice expressed higher levels of interferon-gamma (IFN-**γ**) vs. control mice, while in AdSC-treated mice, IFN-**γ** and eotaxin expression was shown to be reduced in kidney tissue vs. the latter. A tendency towards lower interleukine-2 (IL-2) and MIP-1**α** expression was also observed for these animals (n = 2–6 for each group). Immunohistochemistry values are expressed as the percentage of area of polarized light ± s.d. Scale bar, 50 **μ**m. Bioplex values are expressed as (μg/mL) ± s.d. *P<0.05, **P<0.01 ***P<0.001

In the clinical setting, studies have correlated a higher incidence of CKI amongst patients with AKI. In accordance, researches demonstrate that FA-induced AKI is able to induce kidney fibrosis, reflecting clinical observations. Therefore, we next aimed towards characterizing folic acid-induced progression of CKI and the consequences of AdSC therapy in this injury. In order to evaluate kidney fibrogenesis, picrosirius red staining of collagen fibers was analyzed through polarized light microscopy and quantified. Analysis showed a rise in tissue fibrosis 4 weeks after folic acid-induced kidney injury. In contrast, this profile was significantly reduced in animals that received AdSC therapy 24 h after FA, demonstrating that stem cells were efficient in preventing collagen deposition in kidney tissue, reducing tissue fibrosis at long term (Fig 3A and 3B). In order to better elucidate the long-term effects of AdSC in kidneys, we also performed tissue protein expression assays in the chronic model. Bioplex analyses showed elevated expression of INF-**γ** in kidneys which puts to evidence the chronic character of tissue damage. Moreover, AdSC-treated mice displayed significant reduction in interferon gamma (IFN-**γ**) and eotaxin levels (Fig 3C). These data confirm that AdSC therapy was also capable of halting the persisting inflammatory process even 4 weeks after initial kidney damage.

## Discussion

Stem cell research has received a great amount of attention in the last decade. Due to its immunomodulatory and regenerative properties, its potential has been verified in many different injury models of inflammatory nature. Mesenchymal stem cells in special have been brought into focus for their therapeutic and immunomodulatory role on many diseases as heart injury, type 1 diabetes and kidney injury. In the kidney, various studies have reinforced these positive results, including models as kidneys ischemia/reperfusion injury and toxic insult-related damage, as by cisplatin [10,18]. AdSC, in specific have surged as a feasible source of stem cells not only for research purposes but also clinically due to its accessibility and accentuated immunomodulatory potential. Our studies aimed to characterize the therapeutic role of AdSC in a mouse model of folic acid-induced AKI and to verify the effects of this treatment in the progression of kidney fibrosis.

Our data demonstrate a significant protective role for AdSC treatment against folic acid-induced AKI, both in function and in inflammation pathways. A number of studies corroborate the effectiveness of different types of stem cells on folic acid-induced nephrotoxic injury [19,20]. In addition, most published data suggest that the major mechanism of action of stem cell therapy is regulation of the inflammatory process that develops during injury [21]. As little is known on the predominant immune cell types involved in folic acid-induced kidney injury, we therefore investigated the participation of inflammation in this injury. In this study, we demonstrate that neutrophils posses a major role in folic acid-induced nephrotoxicity. Our data suggests this is mainly due to chemokine-mediated homing to tissue. Neutrophil cell indirect count was also significantly reduced after stem cell treatment, indicating injury resolution and reduced inflammation. These data were corroborated by reduced expression of CXCL-1 and GM-CSF in tissue. Neutrophil invasion has also been described in ischemia/reperfusion injury, and a recent study performed by Doi and collaborators [7] confirm our findings, by observing amelioration of folic acid-induced kidney injury in mice submitted to neutrophil depletion.

Although there are vast data in literature over the acute effects of stem cell treatment, few studies focus on the long-term consequences of this therapy. Folic acid-induced AKI is also a commonly used model to study the progression to CKI, due to fibrogenesis in kidneys. Hence, due to the acute effects seen with AdSC therapy, we postulated that acute AdSC treatment could prevent progression to tissue fibrosis at long-term. We also aimed towards clarifying the possible contra lateral effects of AdSC treatment in folic acid-induced CKI. Although chronic injury induced by FA in mice did not display renal function impairment (data not shown), kidney tissue expressed significantly higher levels of fibrosis. In addition, our data demonstrated that stem cell treatment was capable of preventing chronic collagen tissue deposition. Surprisingly, even 4 weeks after treatment, this finding was accompanied by reduced expression of the chemoattractant eotaxin, and INF-**γ**. These results therefore indicate that acute AdSC treatment was responsible for long-term modulation of the immune response in kidneys. These findings are supported by other studies that also find prolonged immune regulation and reduced fibrosis after stem cell treatment [22,23].

Although some groups have demonstrated a rare presence of stem cells in targeted tissue chronically [14,20], a great majority of studies fail to find these cells in tissue at long-term, including in folic acid-induced injury. Thus, it is plausible to postulate that the effects seen in this study are most probably due to a lasting consequence effect of initial immune system regulation rather than the lasting presence of AdSC in kidneys. In the future, it will be interesting to characterize and compare the immune profile of existing cells during acute and chronic inflammation to determine if stem cell treatment induced the development of a less inflammatory infiltrate. Future studies should also identify the specific molecules responsible for the immune regulation seen in this model.
